# Sensitivity of some nitrogen fixers and the target pest *Fusarium oxysporum* to fungicide thiram

**DOI:** 10.2478/v10102-012-0005-6

**Published:** 2012-03

**Authors:** Awad G. Osman, Ashraf M. Sherif, Adil A. Elhussein, Afrah T. Mohamed

**Affiliations:** 1 Biofertilization Department, Environment and Natural Resource Research Institute, National Center for Research, Sudan; 2 Botany Department, Faculty of Science, University of Khartoum, Khartoum, Sudan

**Keywords:** nitrogen fixers, fusarium oxysporum, thiram, toxicology

## Abstract

This study was carried out to investigate the toxic effects of the fungicide thiram (TMTD) against five nitrogen fixers and the thiram target pest *Fusarium oxysporum* under laboratory conditions. Nitrogen fixing bacteria *Falvobacterium* showed the highest values of LD_50_ and proved to be the most resistant to the fungicide followed by *Fusarium oxysporum*, while *Pseudomonas aurentiaca* was the most affected microorganism. LD_50_ values for these microorganisms were in 2–5 orders of magnitude lower in comparison with LD_50_ value for *Fusarium oxysporum.* Thiram was most toxic to *Pseudomonas aurentiaca* followed by *Azospirillum*. The lowest toxicity index was recorded for *Fusarium oxysporum* and *Flavobacterium*. The slope of the curve for *Azomonas*, *Fusarium oxysporum* and *Flavobacterium* is more steep than that of the other curves, suggesting that even a slight increase of the dose of the fungicide can cause a very strong negative effect. Thiram was more selective to *Pseudomonas aurentiaca* followed by *Azospirillum*, *Rhizobium meliloti* and *Azomonas*. The lowest selectivity index of the fungicide was recorded for *Falvobacterium* followed by *Fusarium oxysporum*. The highest safety coefficient of the fungicide was assigned for *Flavobacterium*, while *Pseudomonas aurentiaca* showed the lowest value.

## Introduction

Pesticides are used for the welfare of human beings but in time they will challenge us by showing their toxicity. We can be exposed to them directly, or indirectly through the food chain. Pesticides are toxic compounds to all living organisms, however the effects vary from species to species. Their excessive use causes serious damage to the ecosystem – terrestrial as well as aquatic, and consequently to the surrounding flora and fauna (Paliwal *et al.,* 2009).

Thiram (Tetramethylthiuram disulfide) is a non-systemic seed dressing fungicide that belongs to the ethylene bisdithiocarbamate (EBDC) chemical class. It is one of the most widely applied dithiocarbamate fungicides in modern agriculture for controlling, damping-off diseases, apple scab, brown rot of stone fruit, Botrytis rot, turf disease, onion smut. It is also used as a seed disinfectant for many vegetables, fungal diseases on safflower, black root of sugar beet, grey mould of strawberries, Botrytis blight in tulips, Colletotrichum lint on flax, for protection of forest nursery seedlings against damping-off and as repellent against rabbit rodents, deer and blackbirds (Montegomery *et al.* 1936; Harrison, 1961; Muskett & Colhoun, 1940; Harrington, 1941; Newhall, 1945; Taylor & Ruppert, 1946; McKeen, 1950; Hildebrand *et al.* 1949; Hildreth & Brown, 1955).

Horsfall (1956) reported that the relationship of thiram to enzyme systems has provided an area of fundamental investigation. He was the first to propose that the fungicidal effect of thiram was connected with its ability to form complexes with heavy metal ions. It was observed that fungitoxicity of TMTD was not reversed by addition of the trace metals Fe, Zn, Cu, Mn and Mo to the medium.

Fungicides were found to have the largest inhibition effect on soil microorganisms (Kruglov, 1991). Many practices used for legume production include inoculation of seeds with rhizobia and treatment of the seeds with fungicides to reduce seed rot and seedling damping-off resulting from infection by soil-borne pathogens (Schroth & Hildebrand, 1964). However, many fungicides are toxic to rhizobia (Diatloff, 1970; Hofer, 1958), and some reduce the amount of N_2_ fixed (Fisher, 1976; Staphorst, & Strijdom, 1976). Thus, seed protection and seed inoculation are frequently incompatible. One way of allowing for successful infection of legume roots with *Rhizobium* after treatment of seeds with fungicides is to use a fungicide-resistant inoculant (Odeyemi & Alexander, 1977). Ogunseitan & Odeyemi (1985) suggested that in the chemical control of pests it is important to avoid serious injury to a great variety of microbes whose functions are vital to the crop-producing power of the soil. Odeyemi & Alexander (1977) reported that treatment of legume seeds with Thiram, Spergon and Phygon before rhizobial inoculation decreased the weight of plants and nitrogen fixation considerably. Lennox & Alexander (1981) reported that application of thiram to seeds inoculated with a thiram-resistant strain of *Rhizobium* resulted in a significant increase in dry weight and nitrogen contents of plants compared with inoculation or thiram treatment alone.

The aim of this study was to evaluate the toxic effect of the fungicide thiram on some soil beneficial microbes with special emphasis on nitrogen fixers, besides testing the efficiency of the fungicide on controlling the target pest.

## Materials and methods

### Source of Thiram

Thiram (TMTD) (25% DP) C_6_H_12_N_2_S_4_ (Mwt: 240.4) was obtained from El Dali and El mazmoum Co. Ltd. Khartoum, Sudan.

### Nitrogen Fixing Bacteria and Fungi Studied

*Azomonas sp*, *Azospirillum sp*, *Flavobacterium sp*, *Pseudomonas aurentiaca*, and *Rhizobium meliloti*, were obtained from the microbiological collection of the Department of Biofertilization of the Environment and Natural Resources Research Institute (ENRRI, Sudan).

*Fusarium oxysporum*, was obtained from the microbiological collection of the Department of Biological Control of the Environment and Natural Resources Research Institute (ENRRI).

### Culture Media Used

Two different media meat peptone agar and Czapek Dox agar, were prepared by dissolving the ingredients of each (g) in one liter of distilled water as follows (Tepper *et al.,* 1993): **Meat Peptone Agar (MPA**)**:** Meat extract 5.0; Peptone 7.5; Sodium chloride 5.0 and Agar 20.0. **Czapek Dox Agar (CZA):** Sucrose 20.0; Sodium 2.0; Dipotassium hydrogen phosphate 1.0; Magnesium sulphate, hydrated (MgSO_4_ .7H_2_O) 0.5; Potassium chloride 0.5; Calcium carbonate 3.0 and Agar 20.0.

### LD_50_ Determination

The concentrations of the fungicide that caused 50% destruction of the cells of pure cultures of the microorganisms (LD_50_) were calculated by log-dose/probit regression line method Finney (1971) using computer software (Biostat, 2008).

A preliminary experiment was conducted to determine thiram effective concentration limits (20–80%) for *Azomonas sp*, *Azospirillum sp*, *Flavobacterium sp*, *Pseudomonas aurentiaca* and *Rhizobium meliloti* as suggested by Zinchenko *et al.* (1974). Each bacterium strain was grown on meat peptone broth for 24 hours. The amount of 0.5 ml of this culture broth was transferred and used to inoculate plates of meat peptone agar supplemented with different thiram concentrations. The plates were incubated at 28 °C for 48 hours and then the colonies present were counted. A control set of MPA plates not supplemented with thiram was prepared for comparison. The inhibition index for each strain was calculated by subtracting the number of colonies counted for the thiram amended plates from the number of colonies recorded for the control plates. The inhibition index so obtained was used to calculate thiram LD_50_ for each strain obtained.

For determining thiram effective concentration limits for *Fusarium oxysporum,* the fungus was grown onto CZA plates for one week and 1.1cm discs were then cut and seeded onto the surface of CZA plates supplemented with different thiram concentrations. A control set in which the fungal discs were seeded onto CZA plates not supplemented with thiram was included. Ten days later, the growth diameters in the treated and control plates were measured and recorded in cm (Shattock, 1988). The index of inhibition was calculated by subtracting the growth diameter recorded for thiram amended plates from those recorded for the control. The value was then used to calculate thiram LD_50_ for *Fusarium oxysporum*.

The calculated LD_50_ for each strain was used to determine the thiram selectivity Index (SI) and safety coefficient (SC) (Kruglov, 1991) as follows:

Selectivity Index:

$$\frac{{\text{LD}}_{50}\;\text{of the first Microorganism}}{{\text{LD}}_{50}\;\text{of the second Microorganism}}$$

Safety coefficient:

$$\frac{{\text{LD}}_{50}}{\text{Field dose}(\text{0.0005719}\;g(\text{ai})/100\text{0.0005719}\mathrm{g soil})}$$

Toxicity index of thiram was determined according to Sun (1950).

## Results

### Effects of Thiram on pure cultures of some N_2_ fixers and *Fusarium oxysporum*

**Figure 1:**
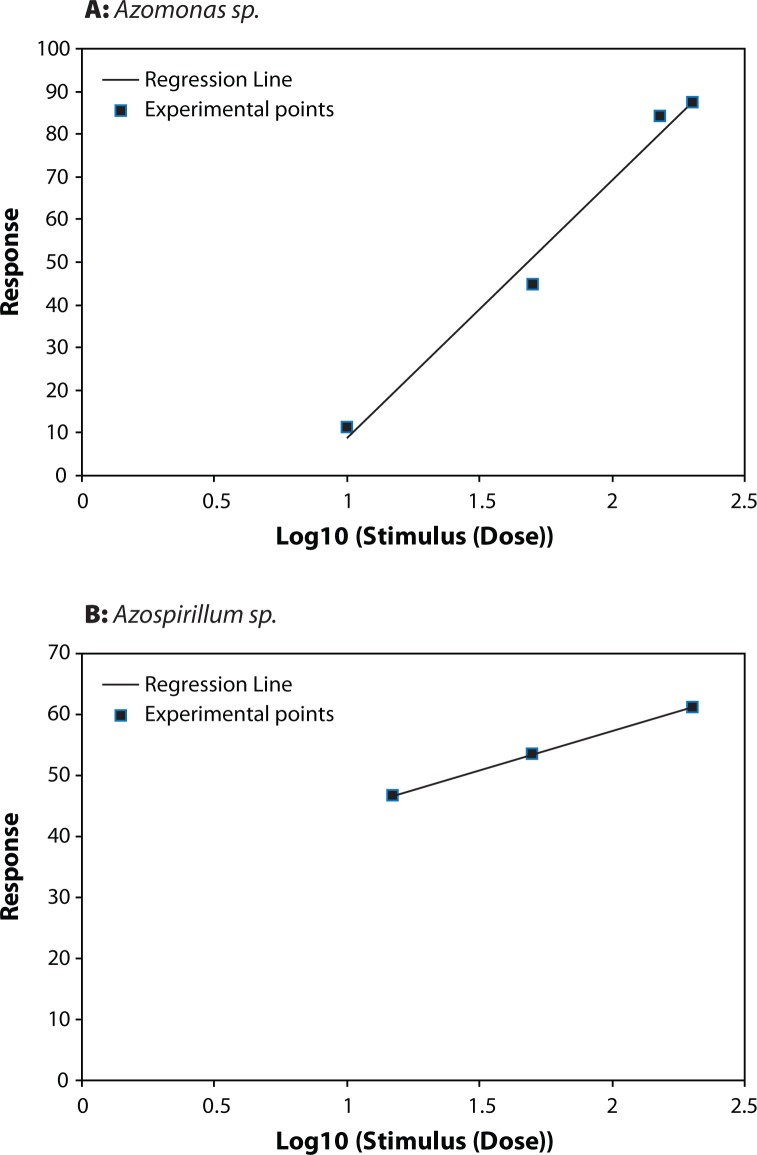
Dose–Effect Curve for (**A**) *Azomonas sp* and (**B**) *Azospirillum sp.*

**Figure 2:**
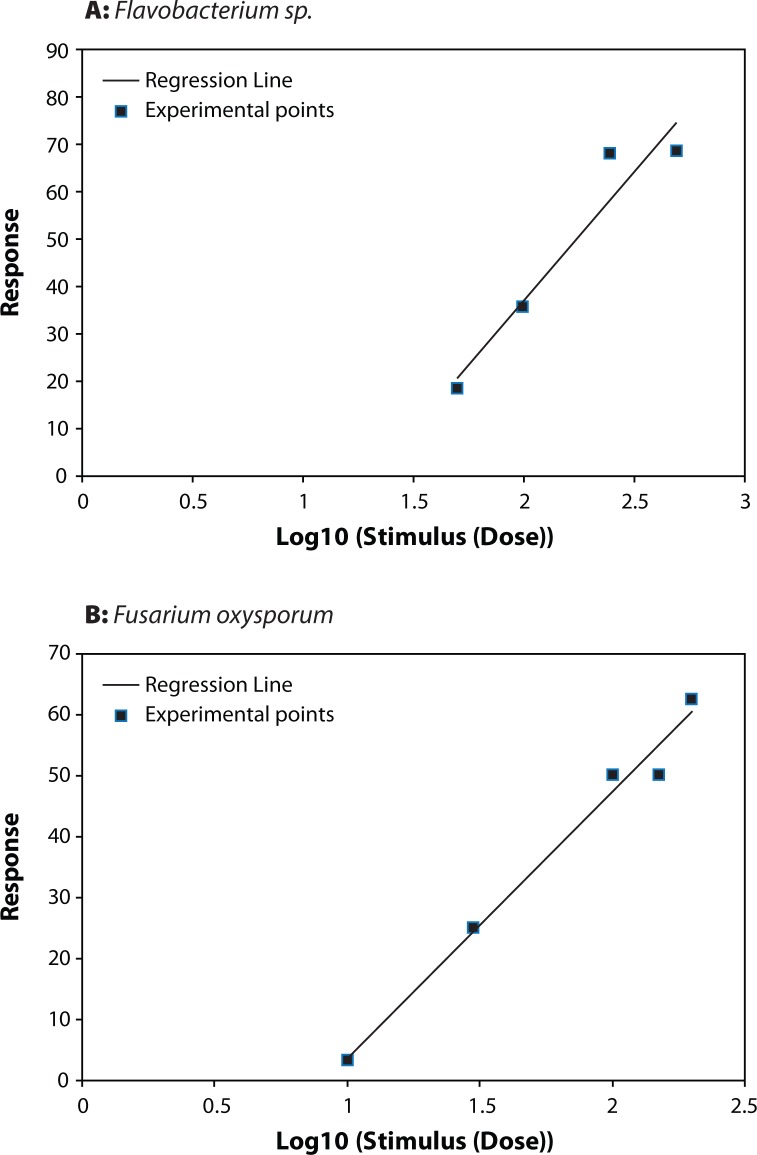
Dose–Effect Curve for (**A**) *Flavobacterium sp* and (**B**) *Fusarium oxysporum*.

**Figure 3:**
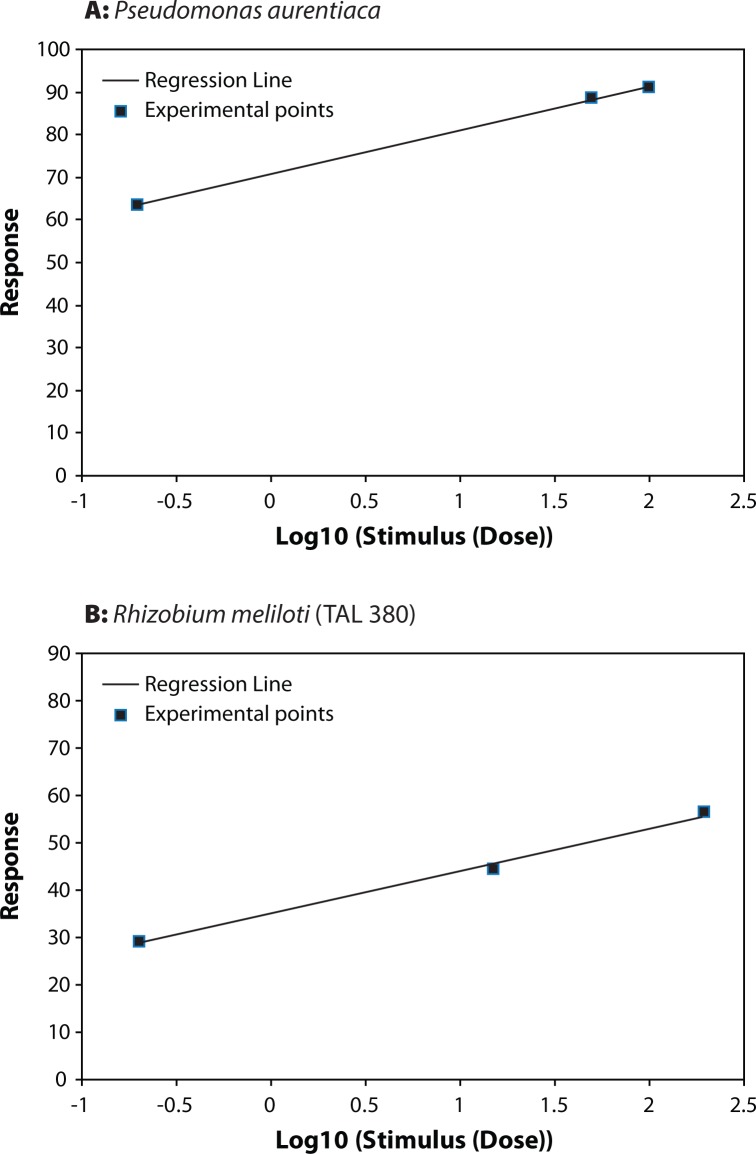
Dose–Effect Curve for (**A**) *Pseudomonas aurentiaca* and (**B**) *Rhizobium meliloti*.

**Plate 1:**
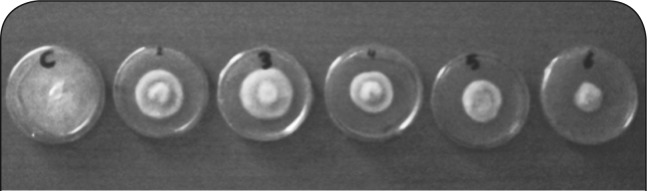
Thiram dose effect (probit analysis) for *Fusarium oxysporum*.

The results of studying the influence of the fungicide thiram upon growth and development of pure cultures of soil bacteria (N_2_ fixers) and *Fusarium oxysporum* are presented in Tables 1 and 2, Figures (1–3) and Plate 1.

**Table 1:** Effect of Thiram on pure cultures of different microorganisms.

		Index of Selectivity					
		1	2	3	4	5	6
Species	LD_50_ (ppm)	*Falvo*	*F. oxysporum*	*Azomonas*	*R. meliloti*	*Azospirillum*	*P. aurentiaca*
*Falvobacterium*	44.685		1.496	3.571	3.957	5.500	7447.5
*F. oxysporum*	29.867			2.387	2.645	4.344	4977.917
*Azomonas*	12.515				1.108	1.820	2085.833
*R. meliloti*	11.292					1.643	1882.083
*Azospirillum*	6.875						1145.833
*P. aurentiaca*	0.006						

**Table 2:** Inhibition of growth of different microorganisms by Thiram.

No	Microorganisms	LD_50_ (ppm)	Safety Coefficient	Toxicity Index (%)
1	*Falvobacterium sp.*	44.685	78134.289	0.0134
2	*F.oxysporum sp.*	29.867	5224.165	0.0201
3	*Azomonas sp.*	12.515	21883.196	0.0479
4	*R. meliloti*	11.292	19744.710	0.0531
5	*Azospirillum sp.*	6.875	12021.332	0.0873
6	*P. aurentiaca*	0.006	10.491	100

*Azomonas*, *Flavobacterium*, *Rhizobium meliloti*, *Pseudomonas aurentiaca*, *Azospirillum* and *Fusarium oxysporum* showed different resistance to thiram with selectivity indexes (SI) in the range of 1.496–7447.5 (Table 1).

The highest LD_50_ (44.685) was recorded for *Falvobacterium* followed by *Fusarium oxysporum*, *Azomonas* and *Rhizobium meliloti*. *Azospirillum* and *Pseudomonas aurentiaca* were the most affected as they recorded the lowest LD_50_ of 6.875 and 0.006 respectively.

Table 1 shows the Index of Selectivity for the different organism tested. It seems quite evident that thiram is more selective to *Pseudomonas aurentiaca,* followed by *Azospirillum*, *Rhizobium meliloti* and *Azomonas*. The lowest Selectivity Index was recorded for *Falvobacterium* and *Fusarium oxysporum*. The highest safety coefficient 78134.289 was signed for the associated nitrogen fixing bacteria *Flavobacterium*, while *Azomonas* showed a low safety coefficient value (Table 2). The toxicity index depending on LD_50_ values of thiram on *Azomonas*, *Flavobacterium*, *Rhizobium meliloti*, *Pseudomonas aurentiaca*, *Azospirillum* and *Fusarium oxysporum* is shown in Table 2. Thiram was most toxic to *Pseudomonas aurentiaca* with toxicity index (100), followed by *Azospirillum*. The lowest toxicity index was recorded for *Fusarium oxysporum* (0.0201) and *Flavobacterium* (0.0134).

## Discussion

The fungicide did not kill the target organism *Fusarium oxysporum* at the concentrations tested in the in vitro experiment, but it was most toxic to the fungus and significantly reduced its growth rate and final colony size at 10 ppm or greater concentrations compared to growth on an amended zapek Dox medium (Figure 2, Plate 1). This may be attributed to the fact that *Fusarium oxysporum* was isolated from a soil that had a history of repeated application of the pesticides particularly the fungicide thiram. Fravel *et al.* (2005) found that at concentrations of 10, 30, 50 or 100 ppm a.i. the fungicide thiram did not kill *Fusarium oxysporum* strain CS-20 in the in vitro experiment, but it was most toxic to the fungus and significantly reduced its growth rate and final colony size at 30 ppm or greater.

Figures (1–3) show that for *Azomonas*, *Azospirillum Flavobacterium*, *Fusarium oxysporum*, *Pseudomonas aurentiaca*, and *Rhizobium meliloti* the dependence of the biological effect of the fungicide on its concentration is very similar as for the angle of inclination, and correspondingly, the rate of rise of the effect. At the same time, the slope of the curve for *Azomonas*, *Fusarium oxysporum and Flavobacterium* is more steep than that of the other curves, suggesting that even a slight increase of the dose of the fungicide can cause a very strong negative effect. Kalinin *et al.* (2002) found that the slope of the dose-reaction curve for *Klebsiella planticola* was more steep than that of the curves of *Pseudomonas putida*, *Azotobacter chrococcum* and *Clostridium acetobutilicum*.

Kalinin *et al.* (2002) found that EC_50_ values for *Pseudomonas putida*, *Klebsiella planticola*, *Azotobacter chrococcum* and *Clostridium acetobutilicum* were in 3–5 orders of magnitude higher in comparison with EC_50_ values for different strains of *Phytophthora infestans* and thus proved to be more resistant to the fungicide azoxystrobin.

Depending on LD_50_ values, thiram was most toxic to *Pseudomonas aurentiaca* with the toxicity index 100. Daoud *et al.* (1990) found that the fungicide benomyl was the most toxic compound against *Alternaria sp* followed by fluazifop and Decis (deltamethrin).

Kalinin *et al.* (2002) found that the selectivity indexes of *Pseudomonas putida*, *Klebsiella planticola*, *Azotobacter chrococcum*, *Clostridium acetobutilicum* and *Phytophthora infestans* were in the range of 13.5–20, indicating that Azoxystrobin had a strong selectivity ability.

The safety coefficient refers to the possibility of the use of microorganisms under test with a specific concentration of the fungicide. From these results we conclude that thiram can be used without any limitations in association with microbial inoculants of biological nitrogen fixers for all the bacteria tested, except the genus *Pseudomonas aurentiaca*. Revellin *et al.* (1993) reported that thiram had a small or no effect on the survival of *Bradyrhizobium japonicum* and on the nodulation and yield of soybeans.

## Acknowledgements

Great thanks and gratefulness to Dr. Hanan Ibrahim – Department of Biological Control – Environment & Natural Resources Research Institute for supporting with fungus pure cultures.
